# Plasma procalcitonin concentrations predict organ dysfunction and outcome in dogs with sepsis

**DOI:** 10.1186/s12917-018-1427-y

**Published:** 2018-03-27

**Authors:** Roberta Troia, Massimo Giunti, Robert Goggs

**Affiliations:** 1 University of Bologna, Bologna, Italy; 2 Department of Clinical Sciences, College of Veterinary Medicine, Cornell University, 930 Campus Road, Ithaca, NY 14853 USA

**Keywords:** Procalcitonin, Sepsis, Septic shock, Multiple organ dysfunction syndrome, Dogs, Biomarker

## Abstract

**Background:**

Procalcitonin (PCT) is a valuable prognostic biomarker in human sepsis that is predictive of organ dysfunction, septic shock and mortality. Data on PCT in dogs is limited. This study aimed to investigate the prognostic value of baseline and serial PCT measurements in dogs with sepsis and to determine the association between PCT and sepsis severity and the presence of organ dysfunction. PCT concentrations were measured in citrated plasma samples collected from 53 dogs with sepsis at the time of admission (T0, *n* = 53) and at 24 h (T1, *n* = 35) and 48 h (T2, *n* = 30) post-admission using a commercial ELISA. Dogs were classified by sepsis severity (sepsis without organ dysfunction; severe sepsis; septic shock) and outcome (survivors; non-survivors). Organ dysfunctions were recorded at T0 and during hospitalization, and the APPLE_fast_ score calculated at T0. Healthy dogs (*n* = 12) were used as controls.

**Results:**

There were 18 septic dogs without organ dysfunction, 24 dogs with severe sepsis and 11 with septic shock. Baseline PCT concentrations were significantly greater in dogs with sepsis compared to healthy controls (*P* < 0.0001), and in dogs with septic shock compared to dogs without cardiovascular compromise (*P* = 0.01). Baseline PCT was significantly correlated with organ dysfunction (*P* = 0.003). Declining PCT concentrations were documented in survivors at T1 and T2 compared to PCT at T0 (*P* = 0.0006), and PCT clearance at 24 h was significantly higher in survivors (*n* = 38) compared to non-survivors (*n* = 15) (*P* = 0.037). Canine APPLE_fast_ score was not predictive of sepsis severity, the development of MODS or outcome.

**Conclusion:**

In dogs with sepsis, PCT concentrations at hospital admissions are predictive of organ dysfunction and septic shock. Serial procalcitonin monitoring may offer valuable prognostic information in canine sepsis, wherein early decreases in PCT concentrations are associated with survival.

**Electronic supplementary material:**

The online version of this article (10.1186/s12917-018-1427-y) contains supplementary material, which is available to authorized users.

## Introduction

Sepsis, the life-threatening organ dysfunction caused by a dysregulated host response to infection [1], is a leading cause of death in critically ill human and veterinary patients [2, 3]. Early diagnosis and prompt recognition of disease severity are crucial to the rapid administration of antimicrobials [4, 5], timely hemodynamic resuscitation [6–8], and to the individualization of care [9]. Thus, there is considerable interest in the development of biomarkers for individual prognostication [10–12], and for the prediction of sepsis-related complications, such as multiple organ dysfunction syndrome (MODS) and septic shock [13–15].

Procalcitonin (PCT), the prohormone of calcitonin, has emerged as a promising sepsis biomarker in humans [16]. Procalcitonin is released ubiquitously in response to microbial toxins and specific pro-inflammatory mediators, and its concentrations rise early after the exposure to an infectious stimulus [17–19]. Several trials and meta-analyses have highlighted the clinical value of PCT for diagnosis of bacterial sepsis in critically ill humans [17]. In addition, PCT has been demonstrated to reliably assess prognosis and to chart the severity of sepsis [20], wherein higher circulating PCT concentrations are present in humans with MODS compared to those without organ dysfunction [21]. Furthermore, although the prognostic significance of a single PCT measurement remains unclear, persistently increased PCT concentrations during hospitalization are associated with increased mortality in humans with severe sepsis, septic shock and MODS [22]. As such, the concept of PCT clearance (PCT-c) may provide superior prognostic value and enhance the study of PCT kinetics [23, 24]. The potential value of monitoring PCT kinetics was demonstrated by a recent multicenter study in humans that identified patients who, by day 4, had failed to clear PCT by more than 80% from baseline had substantially increased mortality [16]. Conversely, circulating PCT concentrations are decreased (and PCT-c is increased) during the recovery phase, when infection control is achieved [16]. As such, monitoring PCT kinetics may aid both the initiation of antimicrobials [25], and the discontinuation of antimicrobials, thereby limiting unnecessary prescribing and reducing the duration of antimicrobial therapy [26–28]. As a result, PCT monitoring is now advocated to guide antimicrobial stewardship in several human systemic infections [29, 30].

Few studies in the veterinary literature have evaluated the ability of biomarkers to predict sepsis severity and MODS occurrence in dogs [31, 32]. In addition, despite the considerable interest in PCT in human medicine, to date few studies have investigated the role of procalcitonin in dogs. In part, this may be the result of the previous lack of an assay for canine PCT protein [33]. Those studies that do exist suggest that in dogs the PCT gene is expressed in extra-thyroidal tissues in a variety of conditions associated with systemic inflammation [34–36]. Plasma procalcitonin concentrations are significantly increased in dogs with sepsis, but there is some overlap in the plasma PCT concentrations found in healthy dogs and those with sepsis (unpublished observations). A preliminary study of PCT mRNA expression in leukocytes in dogs with sepsis suggests that PCT gene expression parallels the course of the underlying disease, however, suggesting that plasma PCT measurements might be of value in monitoring disease progression and the response to therapy in dogs with sepsis [35].

The aims of the present study were therefore: 1) to evaluate associations between plasma procalcitonin concentrations and illness severity, organ dysfunction and outcome in a population of dogs with sepsis; 2) to evaluate the prognostic value of procalcitonin clearance by serial procalcitonin measurements in a population of dogs with sepsis.

## Materials and methods

### Animals

A database of stored plasma samples collected at hospital admission from dogs at-risk for MODS was searched for dogs with sepsis. All dogs were prospectively enrolled between 02/2015 and 04/2017 in a study investigating MODS. That study was approved by the local Institutional Animal Care and Use Committee (University of Bologna DL 26/2014, Project 846) and was undertaken with written informed client consent. Respective primary clinicians determined all aspects of patient management.

Dogs were diagnosed with sepsis if at least 2/4 SIRS criteria were satisfied, as previously described [37], and an infection was confirmed by means of cytology, microbiology, histopathology or real-time polymerase chain reaction. Specifically, in cases of pyometra, the diagnosis was made on the basis of pre-operative ultrasonographic findings, gross evaluation of the uterus during ovariohysterectomy and the results of histopathology [38]. The diagnosis of parvoviral enteritis was based on positive real-time polymerase chain reaction analyses of fecal samples, as previously described [39].

Dogs were eligible for inclusion in the present study if they were hospitalized in the intensive care unit (ICU) for at least 12 h and if an aliquot of citrated plasma collected upon hospital admission was available. All citrate plasma samples were stored frozen at − 80 °C from collection until analysis. Where requested by the attending clinicians, serial blood samples were collected 24 h (T1) and 48 h (T2) after hospital admission. When samples were collected per clinician requests, aliquots of citrated plasma were also frozen at − 80 °C to enable subsequent analyses.

Twelve healthy privately-owned blood donor dogs were enrolled as controls (University of Bologna, Project 581) with informed owner consent. These dogs were eligible if they had no history or evidence of recent or chronic medical conditions and had not received any medication, except for routine preventative healthcare, within the preceding 3 months. Dogs were classified healthy on the basis of history, physical examinations, and complete blood count and serum chemistry results.

### Data collection

As part of the parent MODS study various patient parameters were recorded at the time of hospital admission as follows: medical history including comorbidities and prior treatment, physical examination findings, therapies administered. At hospital admission (T0), non-invasive blood pressure measurement,^1^ and pulse oximetry,^2^ were performed. Blood gas and electrolyte analyses and blood lactate measurement were performed using a point-of-care analyzer.^3^ Complete blood counts and serum chemistry analyses were performed using automated analyzers.^4^^,^^5^ Coagulation assays were performed on citrated plasma using a benchtop automated analyzer.^6^ Patient data were used to calculate the shortened Acute Patient Physiologic and Laboratory Evaluation (APPLE_fast_) score as previously described [40].

### Illness severity and outcome classification

Dogs were classified as suffering from sepsis without organ dysfunction, severe sepsis (sepsis with organ dysfunction) or septic shock based on the Society of Critical Care Medicine consensus definitions from 2001 [41]. Organ dysfunction was deemed present if it was noted at hospital admission or occurred during the course of hospital stay. This organ dysfunction was required to involve an organ system other than that affected by the septic focus e.g. a patient with pneumonia would not be classified as having severe sepsis if only the respiratory system was abnormal. Organ dysfunction criteria were adapted from available canine literature (Table 1). Where appropriate, cutoff values for specific clinicopathologic variables e.g. serum creatinine concentration were based on the upper bound of the reference intervals of the institution laboratory. The number of dysfunctional organs at hospital admission and the maximum number of dysfunctional organs (OD_max_) during hospital stay were also recorded. Outcome was recorded as survival to hospital discharge, death, or euthanasia for disease severity.

**Table 1:** Organ dysfunction criteria adapted for use in the present study

Organ system	Organ dysfunction criterion	Reference
Cardiovascular	Systolic blood pressure < 90 mmHg despite adequate fluid resuscitation	[51]
Hemostatic	PT > 7.5 s OR aPTT > 16.5 s OR Platelet count < 160 × 10^9^/L (< 160 × 10^3^/μL)	[3, 46]
Hepatic	Increase in serum bilirubin > 6.0 μmoL/L (> 0.35 mg/dL) (in the absence of hemolysis or biliary obstruction)	[3, 46]
Renal	Serum creatinine (sCr) > 119.3 μmoL/L (> 1.35 mg/dL) OR Increase in sCr of > 26.5 μmoL/L (≥ 0.3 mg/dL) from baseline OR Oliguria (urine output < 1 mL/kg/h over 6 h)	[52]
Respiratory	SpO_2_ < 95% on room air OR SpO_2_ / FiO_2_ < 315 on supplemental oxygen OR PaO_2_ / FiO_2_ < 400 mmHg on supplemental oxygen	[3, 46, 53]

### Procalcitonin measurements

Plasma concentrations of PCT were measured on all dogs at T0, and on all available samples from T1 and T2 using a commercial ELISA kit.^7^ Procalcitonin concentrations were measured as a batch at Cornell University. Samples from Bologna were transported to Cornell on dry ice by overnight courier. All samples were frozen on arrival and were stored at − 80 °C prior to analysis. Procalcitonin is reportedly stable for prolonged periods at − 80 °C [42]. Procalcitonin clearance (expressed as percent) at 24 and 48 h was calculated according to eq. 1, as previously reported [23, 24].

1 $$ \mathrm{PCT}\ \mathrm{clearance}\ \left(\mathrm{PCT}-\mathrm{c}\right)\ \mathrm{at}\ 24\ \mathrm{h}=\left({\mathrm{PCT}}_{\mathrm{T}0}\hbox{--} {\mathrm{PCT}}_{\mathrm{T}1}\right)/{\mathrm{PCT}}_{\mathrm{T}0}\times 100 $$

### Statistical methods.

Prior to test selection, data were assessed for normality by assessment of histograms, calculation of skewness and kurtosis and with the D’Agostino Pearson test. Descriptive statistics were calculated as appropriate. Most variables were not normally distributed and hence continuous variables are reported as median (min-max) and were compared using non-parametric tests. The Mann Whitney U test and the Kruskal-Wallis test with Dunn’s post-hoc adjustment for multiple comparisons were used to compare continuous variables between groups. Categorical variables were compared using Fisher’s exact test. Scatterplots and calculation of Spearman’s coefficients were used to assess correlations between continuous variables. All analyses were performed using commercial software.^8^ Alpha was set at 0.05.

## Results

### Study population

Fifty-three dogs with sepsis were enrolled. There were 22 intact female dogs, 5 spayed female dogs, 22 intact male dogs and 4 castrated male dogs. The median age was 7y (0.08–15.5). The median bodyweight was 18.5 kg (3.7–55.7). All dogs met at least 2/4 SIRS criteria [37]. The median rectal temperature on presentation was 39.0 °C (36.3–41.0) [102.2 °F (97.3–105.8)], the median heart rate was 135 bpm (60–240), the median respiratory rate was 32 rpm (12–108) and the median leukocyte count was 12.8 × 10^3^/μL (0.37–76.2). The median APPLE_fast_ score was 24 (14–33) and the median duration of hospital stay was 5d (1–20). The causes of sepsis were parvoviral enteritis (*n* = 15), septic peritonitis (*n* = 14), pyometra (*n* = 13), necrotizing fasciitis (*n* = 5); bacterial prostatitis (*n* = 2) and perineal abscess, pyelonephritis, pneumonia and pyothorax (all n = 1). Comorbidities were identified in 21 patients (40%), and included inflammatory-bowel disease, chronic kidney disease, chronic hepatopathy, heart disease, neoplasia, urinary tract obstruction and intestinal parasitism. Thirty dogs received antimicrobials in the 7 days preceding hospital admission, while 2 dogs were receiving immunosuppressive therapy at the time of presentation. Thirty-eight dogs (72%) survived to hospital discharge, while 15 (28%) dogs were non-survivors. Ten of the 15 non-survivors were euthanized. No dog was euthanized due to financial limitations.

Eighteen dogs (34%) had sepsis without organ dysfunction, 24 dogs (45%) had severe sepsis and 11 dogs (21%) showed signs of circulatory failure requiring vasopressors and were classified with septic shock. In the first 24 h of ICU stay kidney and hepatic dysfunction were the most commonly documented *n* = 14 (26%) and *n* = 13 (24%) respectively, followed by cardiovascular *n* = 10 (19%), hemostatic *n* = 9 (17%) and respiratory *n* = 3 (6%). An increase in the number of dysfunctional organs was noted in 17 dogs (35%). It was not possible to determine progression of organ dysfunction in 5 dogs, due to death or euthanasia within 24 h of ICU admission. The median OD_max_ was 1 (0–4). Descriptive statistics for the clinicopathologic variables evaluated in septic dogs are reported in Table 2. The healthy control dog population (*n* = 12) consisted of 5 intact male dogs, 5 intact female dogs and 2 female spayed dogs. The median age was 4y (2–7). The median bodyweight was 29 kg (22–42.4).

**Table 2:** Descriptive statistics for selected clinicopathologic variables measured in dogs with sepsis (*n* = 53). Data are presented as median (min-max)

Variable (units)	Institution reference intervals	Dogs with sepsis (n = 53)
Procalcitonin (pg/mL)	–	103 (15.4–470.2)
APPLE_fast_ score	–	24 (14–33)
Leukocytes (×10^9^/L)	6–17	12.8 (0.37–76.2)
Platelets (×10^9^/L)	160–500	269 (30–602)
Hematocrit (%)	37–55	42.5 (23.7–68.5)
Total protein (g/L)	56–79	60 (27–82)
Albumin (g/L)	28–37	23 (14–35)
Creatinine (μmol/L)	57.5–119.3	75.1 (23.0–686.9)
Bilirubin (μmol/L)	1.2–5.8	3.9 (1.2–50.3)
Glucose (mmol/L)	3.9–6.9	5.4 (0.6–48.3)
Lactate (mmoL/L)	0–2	2.0 (0.5–8.2)
PT (s)	5.0–7.5	6.9 (5–15.7)
aPTT (s)	8.0–16.5	13.1 (9.3–47.1)

### Baseline procalcitonin concentrations

**Fig. 1:**
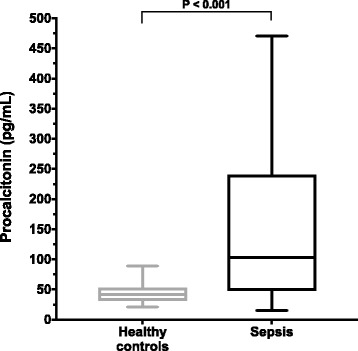
Dogs with sepsis have significantly greater plasma procalcitonin concentrations than healthy controls. A box and whisker plot comparing the plasma procalcitonin (PCT) concentrations from 12 healthy control dogs and 53 dogs with sepsis. The central lines represent the median, the boundaries of the boxes represent the interquartile range and the whiskers represent the minimum and maximum values. Dogs with sepsis had significantly greater plasma concentrations of PCT compared to healthy controls (*P* < 0.001) by Mann-Whitney U test

**Fig. 2:**
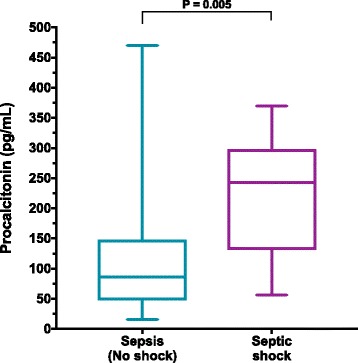
Plasma procalcitonin concentrations are significantly greater in dogs with septic shock compared to dogs without cardiovascular compromise. A box and whisker plot comparing the baseline (T0) plasma procalcitonin (PCT) concentrations in dogs with sepsis but without shock (*n* = 42) and dogs with septic shock (*n* = 11). The central lines represent the median, the boundaries of the boxes represent the interquartile range and the whiskers represent the minimum and maximum values. Dogs with septic shock had significantly greater plasma concentrations of PCT compared to dogs without septic shock (*P* = 0.005) by Mann-Whitney U test

**Fig. 3:**
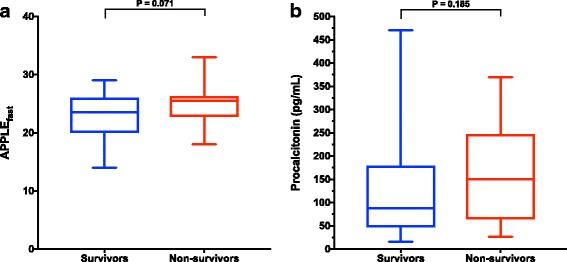
Illness severity and plasma procalcitonin (PCT) concentrations at presentation are not significantly associated with outcome. Box and whisker plots comparing baseline APPLE_fast_ scores (**a**) and plasma procalcitonin concentrations (**b**) between survivors (*n* = 38) and non-survivors (*n* = 15). The central lines represent the median, the boundaries of the boxes represent the interquartile range and the whiskers represent the minimum and maximum values. No significant difference was documented by Mann-Whitney U test for APPLE_fast_ (*P* = 0.071) or PCT concentration (*P* = 0.185) between the two groups

Upon presentation, the median PCT concentration in the dogs with sepsis was significantly greater than in healthy controls 103.0 pg/mL (15.4–470.2) versus 41.6 pg/mL (21.5–88.7) (*P* < 0.001) (Fig. 1). The baseline (T0) PCT concentrations were significantly greater in dogs with septic shock compared to dogs with sepsis but without cardiovascular compromise 243.1 pg/mL (56.7–369.8) vs. 77 pg/mL (18.2–470.2) (*P* = 0.005) (Fig. 2). There was no significant difference in the PCT concentrations at T0 between survivors and non-survivors 87.64 pg/mL (15.39–470.2) vs. 150.2 pg/mL (26.34–369.8) (*P* = 0.18) (Fig. 3a). Procalcitonin concentration at T0 was positively correlated with OD_max_ (r_s_ 0.4, *P* = 0.003) and negatively correlated with leukocyte count (r_s_ − 0.49, *P* = 0.0002) (Additional file 1: Figure S1A). There was no significant difference between the PCT concentrations of dogs with versus without comorbidities (*P* = 0.99), or between dogs that had received antimicrobials prior to presentation compared to those that did not (*P* = 0.59). There was no significant correlation between PCT concentration and APPLE_fast_ score (*P* = 0.26).

### Serial procalcitonin evaluation

**Fig. 4:**
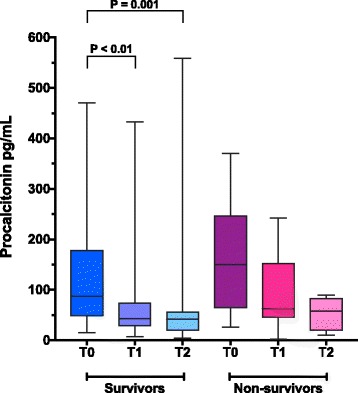
In dogs with sepsis, procalcitonin (PCT) clearance over the first 48 h of hospitalization is significantly greater in survivors than in non-survivors. Box and whisker plots comparing serial plasma PCT concentrations at baseline (T0), + 24 h (T1) and + 48 h (T2) in survivors and non-survivors. The central lines represent the median, the boxes represent the interquartile range and the whiskers represent the minimum and maximum values. Survivors showed a significant decline in PCT concentrations from baseline to T1 (*P* < 0.01) and T2 (*P* = 0.001) by Kruskal-Wallis with Dunn’s post-hoc multiple comparisons test. No statistically significant difference in serial PCT concentrations was detected for non-survivors between any of the time points (overall Kruskal-Wallis *P* = 0.06)

Thirty-eight dogs (72%) underwent serial blood sampling during the first 48 h of ICU stay: there were 35 samples available for PCT measurement at T1, and 30 samples available at T2. In the whole population, baseline (T0) PCT concentrations 103.0 pg/mL (15.39–470.2) were significantly greater than PCT concentrations at T1 45.34 pg/mL (2.02–432.7) (*P* = 0.002) and at T2 42.79 pg/mL (3.9–559.3) (*P* < 0.001) (Additional file 2: Figure S2). Survivors had significantly lower PCT concentrations at T1 42.5 pg/mL (7.5–432.7) (*P* < 0.01), and T2 41.9 pg/mL (3.9–559.3) (*P* = 0.001) compared to respective PCT concentrations at presentation (Fig. 4). There were no significant differences in plasma PCT concentrations between baseline and T1 or T2 in in non-survivors (*P* = 0.06) (Fig. 4). Procalcitonin clearance was significantly higher in survivors compared to non-survivors 44% (− 38 to 82) versus 1% (− 39 to 62) (*P* = 0.037) at 24 h but not at 48 h (*P* = 0.59).

### Clinicopathologic data

Dogs with septic shock had significantly lower white blood cell counts compared to dogs without cardiovascular compromise 4.5 × 10^3^/μL (0.37–29.7) versus 15.1 × 10^3^/μL (0.38–76.2) (*P* = 0.007). Non-survivors had a significantly shorter duration of hospital stay compared to survivors 5d (2–20) versus 3d (1–11) (*P* = 0.049). The number of dysfunctional organs was significantly higher in non-survivors compared to survivors both at the time of admission 0 (0–2) versus 1 (1–4) and during ICU stay 2 (1–4) versus 1 (0–3) (both *P* < 0.0001). At the time of presentation, the frequency of hepatic and of cardiovascular dysfunction was greater in non-survivors compared to survivors 53% versus 13% (*P* = 0.004) and 47% versus 8% (*P* = 0.003) respectively. Absolute frequencies of renal, hemostatic and respiratory dysfunction upon admission were higher in non-survivors, but these differences were not statistically significant. The APPLE_fast_ score at the time of presentation was not significantly different between dogs with distinct sepsis severity classifications (*P* = 0.783), or between survivors and non-survivors (*P* = 0.071) (Fig. 3b).

## Discussion

In the present study dogs with sepsis had significantly greater plasma PCT concentrations than healthy control dogs. Based on the previous veterinary literature [35, 36], and our own observations, this result was expected and suggests that PCT is a potential biomarker for sepsis in dogs. There was some overlap between the concentrations of PCT in septic and healthy dogs, however, which may limit the utility of PCT measurement for the diagnosis of sepsis. The degree of overlap noted in the present study is comparable to our previous observations (unpublished data). As such, canine acute phase proteins, such as C-reactive protein, may be better suited to the identification of systemic inflammation than PCT [43]. The majority of the dogs with comparable concentrations to those of healthy dogs had sepsis without organ dysfunction, suggesting that PCT may be more specific for cases of severe sepsis and septic shock. Other data from the present study suggest that the magnitude of PCT alteration may be related to sepsis severity and organ dysfunction. Plasma PCT concentrations were significantly higher in dogs with septic shock compared to dogs without cardiovascular compromise.

The PCT concentrations in the dogs with sepsis were quite variable (Fig. 1), which accounts for some of the overlap between the healthy controls and the dogs with sepsis. The large range of PCT concentrations in the dogs with sepsis was likely due to variation in illness severity in these dogs. This is supported by the significant difference identified between the PCT concentrations in dogs with sepsis and in those dogs with septic shock (Fig. 2). In humans, the diagnostic accuracy of circulating PCT for sepsis varies with illness severity, wherein PCT is more accurate for identification of severe sepsis and septic shock [17]. This suggests that PCT may be optimally used to assess illness severity in patients that already have a working diagnosis of sepsis. Likewise, PCT measurements might aid with the prediction of impending septic shock or the development of organ dysfunction.

In the present study, PCT concentrations at the time of presentation were not prognostic. This finding may result from an inherent limitation in the discriminant ability of PCT in canine sepsis. It might also be due to the bias of early therapeutic interventions that can positively influence a patient’s outcome. Similar findings have been reported for several acute phase proteins in dogs, wherein markedly abnormal baseline values do not necessarily herald a poor prognosis [44, 45]. The present study suggests that baseline PCT concentrations are correlated with the development and with the degree of organ dysfunction and with the presence of septic shock. These abnormalities themselves are associated with non-survival in dogs with sepsis, however [3, 46]. As such, it may be that the lack of association between baseline PCT and outcome here relates to limited sample size and a low illness severity in the overall population. It may be that the optimal use for a baseline PCT measurement is the identification of patients at risk for later complications. This would be valuable information for clinicians, who might be able to adjust or intensify therapy, to provide additional monitoring for high-risk patients and to prompt early screening to identify organ dysfunction.

The present study found no correlation between baseline PCT concentration and the APPLE_fast_ score. This suggests that these two indices provide different information about dogs with sepsis. Although the prognostic value of the APPLE_fast_ score has been reported in previous studies of critically ill dogs [40, 47–49], it was not predictive of outcome in the present population, nor was it associated with the occurrence of MODS. Individual illness severity scores are not universally predictive, however, and a lack of prognostic value has been reported in other clinical settings [48]. It may be that combinations of illness-severity scores and biomarkers or combinations of biomarkers may be needed to accurately prognosticate complex clinical syndromes like sepsis [49, 50].

In the present study, a significant negative correlation was documented between baseline PCT and leukocyte count, which may point to PCT being an early marker of disease severity in canine sepsis. Dogs in the present study with septic shock had higher PCT concentrations and lower leukocyte counts. Most of the dogs with septic shock in the present study (7/11 dogs) were diagnosed with parvoviral enteritis. This may have accounted for the lower leukocyte counts identified in this sub-group. Interestingly, no differences in PCT concentrations was detected between groups classified by sepsis cause in the present study (data not shown).

The present study suggests that evaluation of PCT kinetics may be a promising prognostic tool for dogs with sepsis. Significantly decreased PCT concentrations from baseline and a high PCT clearance at 24 h were documented in survivors. This suggests the early downregulation of PCT expression in patients recovering from sepsis. In contrast, PCT clearance was low in non-survivors, suggesting that persistently increased PCT concentrations should alert clinicians to the possibility of treatment failures, novel infections or persistent organ dysfunction. This parallels the situation in people, where a lack of reduction in PCT concentrations from baseline is a strong predictor of mortality in sepsis [20], and specific cut-off values have been validated to aid prognostication in individuals [16].

The present study adds to our understanding of the links between biomarkers and MODS and to the association of MODS with outcome in canine sepsis. The number of dysfunctional organs both at the time of admission and during hospital stay, as well as the magnitude of organ dysfunction, were significantly higher in non-survivors. These results corroborate the findings of two recent studies in critically ill dogs documenting a strong relationship between the severity of organ dysfunction and mortality [3, 46], and further advocate for systematic and frequent screening for MODS in critically ill dogs.

The present study may have some limitations. Although septic dogs were enrolled prospectively for the parent MODS study, PCT analyses were performed retrospectively on the citrated plasma samples available. Thus, complete and consistent serial evaluation of PCT concentrations was not possible for the whole study population. In addition, the relatively low number of non-survivors, as well as the occurrence of early death or euthanasia (< 24 h of ICU stay) may have diminished statistical power, leading to an underestimation of the prognostic significance of PCT clearance. Although euthanasia was performed only after clinical judgement of moribund condition or end-stage disease, its impact should be taken into account as a potential source of bias. Finally, the study population was heterogeneous in terms of onset and causes of sepsis, treatment protocols and illness severity. Studies focusing on selected septic diseases and on more severely affected septic patients exclusively (e.g. severe sepsis and septic shock) might better enable investigation of the utility of PCT measurements [17, 23]. The results of the present study are likely to too preliminary to be used to guide alterations to the diagnostic or management plans for individual dogs with sepsis. Confirmation of these findings in other populations will be necessary before clinicians should consider incorporating PCT measurement into their decision-making. Finally, the ELISA method used to measure canine PCT in the present study is currently only accessible in a research setting and a point-of-care test is not currently available. The routine availability of PCT measurement would be necessary to make PCT guided therapies possible in veterinary medicine.

## Conclusions

In conclusion, the present study suggests that PCT may be a promising prognostic biomarker in canine sepsis, specifically that baseline procalcitonin concentrations are associated with the development of MODS and with septic shock, while PCT clearance in the first 24 h is associated with survival and recovery from sepsis. Further prospective studies are necessary to confirm the prognostic significance of serial procalcitonin measurement in distinct populations over longer time-intervals. Future studies might also compare the diagnostic and prognostic utility of PCT with that of other biomarkers in order that biomarker guided therapeutic strategies may become possible.

## Additional files

Additional file 1: **Figure S1.** Scatterplots and Spearman’s coefficients assessing correlations between baseline procalcitonin (PCT) concentrations and the maximum number of organ dysfunction (OD_max_), leukocyte count and the APPLE_fast_ score. Plasma PCT concentrations are significantly and positively correlated with OD_max_ and inversely correlated with leukocyte count (r_s_ 0.400, *P* = 0.003; r_s_ − 0.488, *P* = 0.0002 respectively). There was no significant correlation between plasma PCT and the APPLE_fast_ score (r_s_ 0.126; *P* = 0.2601). (EPS 139 kb)

Additional file 2: **Figure S2.** In hospitalized dogs with sepsis managed with clinician-driven standard care, there is a significant clearance of baseline procalcitonin (PCT) concentrations over time. Box and whisker plots comparing serial plasma PCT concentrations at hospital admission (T0), and + 24 h (T1) and + 48 h (T2) in 53 dogs with sepsis. The central lines represent the median, the boxes represent the interquartile range and the whiskers represent the minimum and maximum values. There are significant decreases in PCT concentrations from T0 to T1 (*P* = 0.002) and T2 (*P* < 0.001) by Kruskal-Wallis with Dunn’s post-hoc multiple comparisons test. (EPS 69 kb)

## Abbreviations

APPLE: acute patient physiologic and laboratory evaluation
ELISA: enzyme-linked immunosorbent assay
ICU: intensive care unit
MODS: multiple organ dysfunction system
PCT: procalcitonin
SIRS: systemic inflammatory response syndrome

## Footnotes

1 petMAP graphic, Ramsey Medical Inc., Tampa, FL

2 Nellcor SpO_2_ module for Dash 3000, GE Healthcare, Rahway, NJ

3 ABL800 FLEX, Radiometer Medical ApS, Denmark

4 ADVIA 2120, Siemens Healthcare Diagnostics, Tarrytown, NY

5 Olympus AU 400, Olympus/Beckman Coulter, Brea, CA

6 BFT II Analyzer, Siemens, Germany

7 Canine Procalcitonin ELISA, Biovendor LLC, Asheville, NC

8 Prism 7.0, GraphPad, La Jolla, CA
